# Dilemmas in the management of screen-detected lesions in patients at high risk for pancreatic cancer

**DOI:** 10.1007/s10689-016-9915-3

**Published:** 2016-07-12

**Authors:** Isaura S. Ibrahim, Bert A. Bonsing, Rutger-Jan Swijnenburg, Lieke Welling, Roeland A. Veenendaal, Martin N. J. M. Wasser, Hans Morreau, Akin Inderson, Hans F. A. Vasen

**Affiliations:** 1 Department of Gastroenterology and Hepatology, Leiden University Medical Centre, Albinusdreef 2, 2333 ZA Leiden, The Netherlands; 2 Department of Surgery, Leiden University Medical Centre, Leiden, The Netherlands; 3 Department of Radiology, Leiden University Medical Centre, Leiden, The Netherlands; 4 Department of Pathology, Leiden University Medical Centre, Leiden, The Netherlands

**Keywords:** Pancreatic ductal adenocarcinoma, PDAC, Genetic predisposition, *CDKN2A* mutation, *p16*-*Leiden* mutation, Surveillance, Pylorus-preserving pancreaticoduodenectomy, Complications

## Abstract

In 3–5 % of all cases of pancreatic ductal adenocarcinoma (PDAC), hereditary factors influence etiology. While surveillance of high-risk individuals may improve the prognosis, this study describes two very different outcomes in patients with screen-detected lesions. In 2000, a surveillance program of carriers of a *CDKN2A/p16*-*Leiden*-mutation consisting of annual MRI was initiated. Patients with a suspected pancreatic lesion undergo CT-scan and Endoscopic Ultrasound, and surgery is offered when a lesion is confirmed. In 2015, two patients with a screen-detected solid lesion were identified. In both patients, lesions were visible on MRI and CT scan, while the EUS was unremarkable. Surgical resection of the head of the pancreas resulted in nearly fatal complications in the first patient. This patient was shown to have a benign lesion. In contrast, timely identification of an early cancer in the second patient was accompanied by an uneventful postoperative course. These cases underline the risks inherent to a PDAC prevention program. All patients should be fully informed about the possible outcomes before joining a surveillance program.

## Introduction

Pancreatic ductal adenocarcinoma (PDAC) is considered one of the most aggressive forms of cancer. With PDAC currently ranking fourth in terms of cancer-related deaths in the United States [1], the prognosis will only improve if the tumour can be detected and treated at an earlier stage.

Approximately 3–5 % of all patients with PDAC have a genetic predisposition that results in an increased risk of developing the tumor [2] and a substantial proportion of these patients carry an underlying gene defect in *CDKN2A/p16*-*Leiden* (Familial Atypical Multiple Mole Melanoma, FAMMM syndrome), *STK11* (Peutz-Jeghers syndrome), the *BRCA1/2* genes (Hereditary breast cancer) or one of the *MMR* genes (Lynch syndrome) [3].

Because surveillance might improve the prognosis in asymptomatic, high-risk individuals, in 2000 a surveillance program for *CDKN2A/p16*-*Leiden* mutation carriers was initiated at the department of Gastroenterology and Radiology at the Leiden University Medical Centre (LUMC). Surveillance consists of a yearly MRI, with an option for EUS between two MRI scans. In cases where a pancreatic lesion is suspected, an EUS and CT scan is performed in order to confirm the presence of the lesion. If the lesion is confirmed, pancreatic surgery is offered.

In this report, we describe surveillance and treatment results for two *CDKN2A/p16*-*Leiden* patients with a screen-detected lesion.

## Case 1

The first patient, a 55-year-old male with a *CDKN2A/p16*-*Leiden* mutation, was referred to the Department of Gastroenterology and Hepatology at the Leiden University Medical Centre in 2011 to discuss the option of pancreatic surveillance. The patient had no known family history of PDAC, and quit smoking in 2003.

**Fig. 1:**
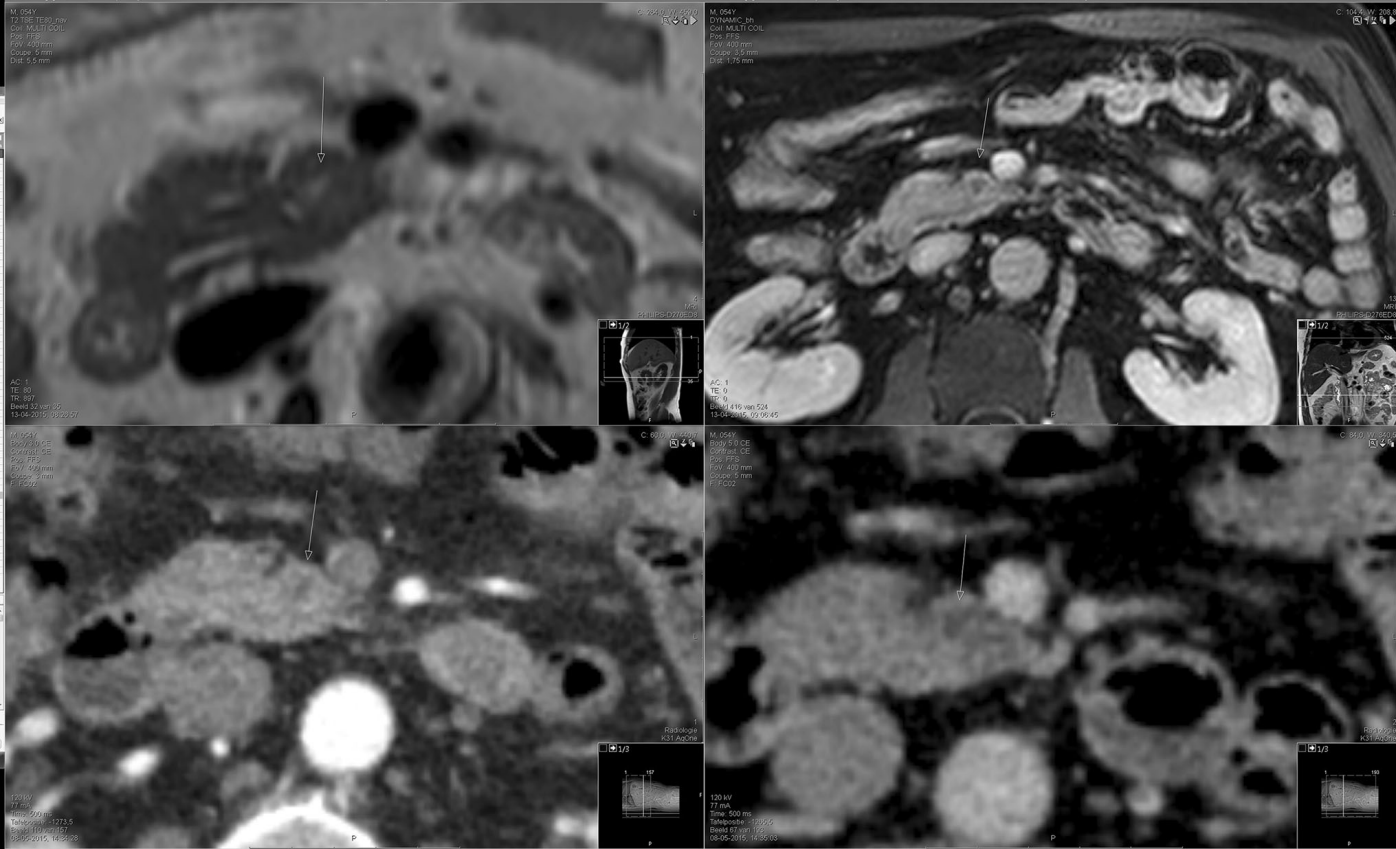
MRI (*upper panel*) and CT-scan (*lower panel*) of the pancreas in case 1

The advantages and disadvantages of the surveillance program were discussed with the patient before he gave informed consent. In the summer of 2015, a solid 8 mm lesion in the uncinate process of the pancreas was detected by MRI (Fig. 1, upper panel). Retrospectively, a small lesion was already visible on the previous MRI in 2014. The patient did not report any complaints and all blood tests were normal, including CA19.9. Subsequent CT scanning confirmed the presence of a solid 10 mm lesion (Fig. 1, lower panel), whereas EUS was normal.

The patient was discussed by a multidisciplinary team and resection was recommended because two of the three imaging tools showed the presence of a solid lesion.

During surgical exploration, a small lesion was palpated in the uncinate process of the pancreas and a pylorus-preserving pancreaticoduodenectomy (PPPD) was performed. Pathological examination of the surgical specimen showed a 3 mm small area with sclerotic stroma and inflammation. Amidst the sclerosis ductular proliferation, with focal cribriform architecture was found. SMAD4 and p53 immunostaining was normal. Taking everything into account it was concluded that there was no evidence of (pre)cancer. A total of 23 lymph nodes were identified, all of which were free of tumor.

One day after surgery the patient developed symptoms suggesting leakage of the choledochojenunostomy. During re-exploration the anastomosis was revised. Eight days after the initial surgery, leakage of the pancreatico-jejunostomy led to a re-laparotomy, with revision of the anastomosis with surgical drains left in situ. Nine days after this intervention, the patient’s condition deteriorated. Evidence for a new leakage of the pancreatic anastomosis led to a completion pancreatectomy. Eighteen weeks later, a retroperitoneal debridement of necrosis in the former pancreatic bed was performed. Finally, the patient developed a thoracic empyema and a subphrenic abscess treated by thoracotomy and decortication. Following the last intervention the patient recovered slowly and he was discharged in a relatively good physical condition, 5 months after the initial surgery. His diabetes is currently managed with four daily doses of insulin.

## Case 2

**Fig. 2:**
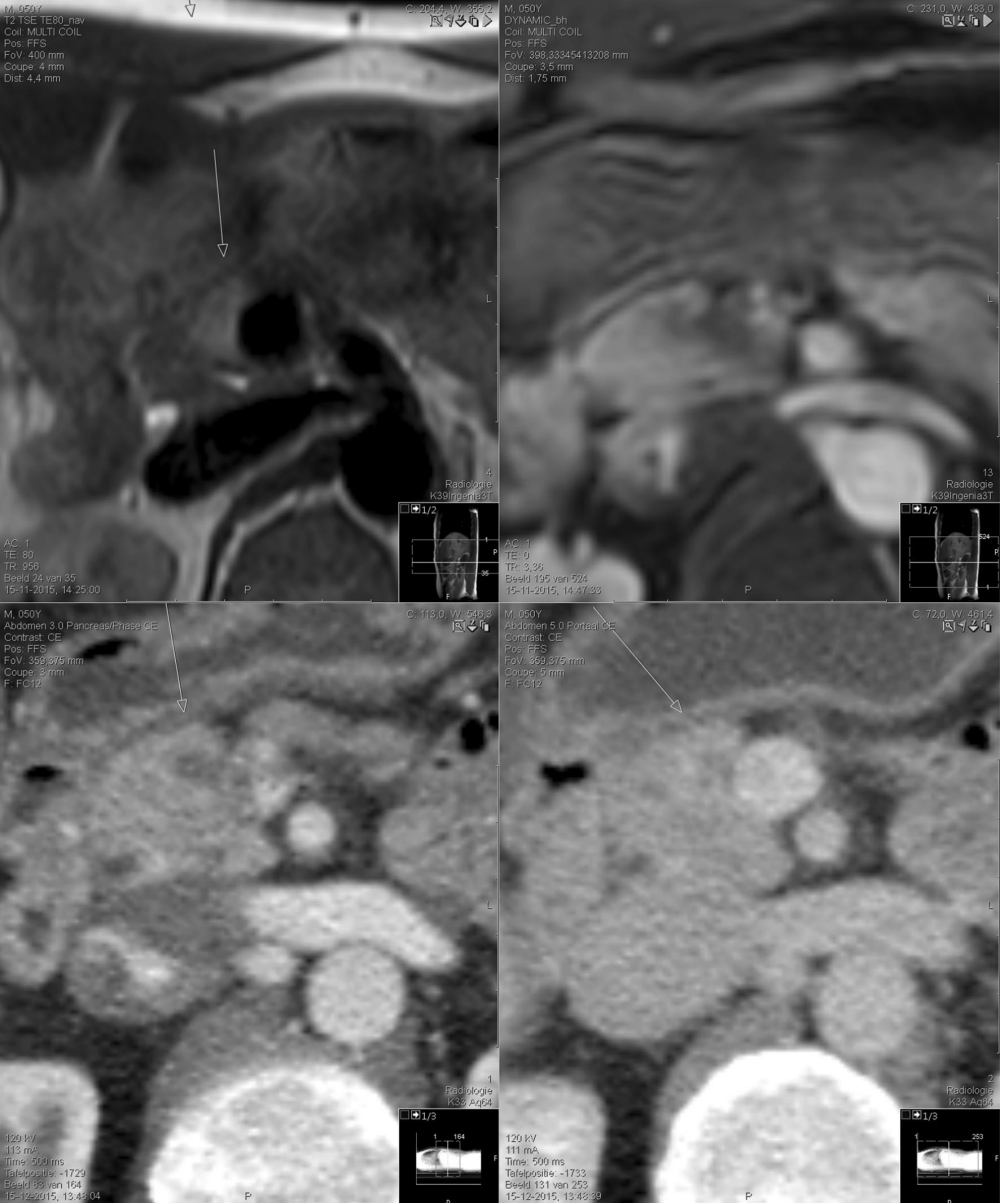
MRI (*upper panel*) and CT-scan (*lower panel*) of the pancreas in case 2

The second patient, a 50-year-old male with a *CDKN2A/p16-Leiden* mutation, was referred to the department of Gastroenterology and Hepatology. He underwent treatment for melanoma at the ages of 36 and 40. He was asymptomatic and he had never smoked. His father died of PDAC at age 52. After discussion on the benefits and drawbacks, the patient decided to participate in the surveillance program (2012). An MRI scan in November 2015 showed a possible 17 mm lesion with oedema in the head of the pancreas (Fig. 2, upper panel). Retrospectively, a smaller oedematous area was present at this site on the previous MRI scan. CT scanning confirmed the presence of a solid 10 mm lesion in the same area (Fig. 2, lower panel), while the EUS was unremarkable. Blood tests did not show any abnormalities. The findings were discussed by the Leiden multidisciplinary team and a PPPD was offered. Following surgery, pathological examination of the surgical specimen showed a 9 mm moderately differentiated PDAC, surrounded by inflammation. The resection margins were free (closed margin 0.3 mm facing the SMV) although there was growth into the peripancreatic tissue. All 15 detected lymph nodes were free of cancer. The patient recovered well after surgery and did not encounter any complications. He was discharged from hospital, in good physical condition, 8 days after initial surgery.

## Discussion

These two cases clearly illustrate the dilemmas faced in the surveillance of individuals at high-risk for PDAC. The first patient experienced nearly fatal complications due to surgery and was found to have a benign lesion. This is an example of a worst-case scenario that may occur in this type of surveillance program. The second patient, diagnosed shortly after the first case, had very similar imaging findings, an uneventful course after surgery, and was eventually shown to have an early cancer.

Several questions arise regarding these two patients: (a) Did the findings, especially in the first patient, justify surgery? (b) Could the benign nature of the lesion in the first patient have been predicted? (c) How can the surveillance programs be improved? (d) How can a devastating course, as seen in the first patient, be prevented?

Regarding the first question, the two imaging techniques (MRI and EUS) reportedly show a high sensitivity and specificity [4], with MRI usually regarded as the best tool to identify cystic lesions and EUS as the best technique for the identification of solid lesions [5]. In both cases reported here the presence of a solid lesion was shown on MRI and CT, whereas the EUS was unremarkable. The fact that the lesion was palpated in both patients during surgical exploration confirmed the imaging findings and justified surgery in view of the high risk of PDAC. Lesion growth is a strong indicator for malignancy, but both patients showed only slight lesional growth. Due to the rapid growth of PDAC, another argument in favour of surgery is the short window of time between the detection of a lesion and development of metastatic disease [6].

In relation to the second issue, prediction of the benign nature of a lesion, differentiation of benign and malignant lesions by FNA biopsy might have been considered. In this particular case no abnormalities were found on EUS, ruling out EUS-guided biopsy. In retrospect, even if the lesion had been visible on EUS, performance of an FNA biopsy would not have been useful in decision-making in this case because a negative FNA result does not exclude the presence of PDAC.

The second patient was diagnosed shortly after discharge of the first patient. In view of the devastating course in the first patient combined with the identification of a benign lesion, we were very hesitant to offer surgery again. However, based on the same arguments and after consultation with international experts, surgery was offered. The pathological findings following surgery in this case subsequently confirmed that this was the right decision and suggested that postponement of surgery would have impaired the patient’s outcome.

Regarding the third question—improvement of surveillance methods—this case report underlines the urgent need for modification of screening methods, especially regarding improvements in the sensitivity of MRI imaging of the pancreas. Additional screening tools should also be developed. At present, the value of the FDG-PET scan in the detection of PDAC is questionable, because the minimum size of lesions detectable by this technique is about 10 mm. However, developments in PET tracers that target specific tumor biomarkers that occur as a consequence of the *CDKN2A/p16*-*Leiden* defect could potentially lead to earlier detection [7].

Another way to improve the surveillance program is the use of circulating tumour markers. Slater et al. [8, 9] reported promising results on the use of tumour markers, including micro-RNAs 196a and b, LCN2, and TIMP1. In a small pilot study, the application of proteomics allowed us to differentiate between malignant and benign lesions [10]. However, these findings should be confirmed in larger studies.

The final question concerns how the risks of serious complications due to surgery can be minimized. Recent studies suggest that mortality rates for pancreaticoduodenectomies procedure lie somewhere between 0.5 and 6 %, with a morbidity rate of up to 40 % [11, 12]. A recent decision model study showed that the possible benefits of a surveillance program may be lost if the mortality rate is slightly increased [13].

The only way to achieve the lowest possible mortality and morbidity rates is to restrict prevention programs to expert centres that carry out larges volume of pancreatic surgeries. Moreover, it is very important to discuss the advantages and disadvantages with a patient prior to their participation in a surveillance program so that the patient is fully aware of the risks. Advantages of the program in *CDKN2A/p16-Leiden* mutation carriers are that more tumours are identified at a resectable stage (75 % vs. 15–20 % in symptomatic patients) and that the prognosis of patients with screen-detected tumours is better (5-year survival is 24 %) than that of symptomatic patients (5–7 %) [14].

Disadvantages include, (1) the surveillance program cannot guarantee that PDAC is always detected at an early and curable stage, (2) the screening protocol is burdensome and may cause anxiety before and shortly after the screening procedure, (3) there may be false positive and false negative cases, and finally, (4) treatment consists of major surgery, a pancreaticoduodenectomy or distal pancreatectomy depending on the site of the tumor, all of which are associated with substantial morbidity and mortality.

These case reports illustrate the difficult decisions that have to be made in high-risk individuals with a suspected lesion in the pancreas. All involved physicians, together with the patient, should be aware of all possible outcomes of the intervention.
